# Mitigating the impact of microbial pressure on great *(Parus major*) and blue (*Cyanistes caeruleus*) tit hatching success through maternal immune investment

**DOI:** 10.1371/journal.pone.0204022

**Published:** 2018-10-04

**Authors:** Roschong Boonyarittichaikij, Elin Verbrugghe, Daan Dekeukeleire, Diederik Strubbe, Sarah Van Praet, Robbe De Beelde, Lieze Rouffaer, Frank Pasmans, Dries Bonte, Kris Verheyen, Luc Lens, An Martel

**Affiliations:** 1 Department of Pathology, Bacteriology and Avian Diseases, Faculty of Veterinary Medicine, Ghent University, Merelbeke, Belgium; 2 Department of Clinical Sciences and Public Health, Faculty of Veterinary Science, Mahidol University, Phuttamonthon, Nakhon Pathom, Thailand; 3 Terrestrial Ecology Unit, Department of Biology, Ghent University, Ghent, Belgium; 4 Forest & Nature Lab, Department of Forest and Water Management, Ghent University, Gontrode, Belgium; Tokat Gaziosmanpasa University, TURKEY

## Abstract

The hatching success of a bird’s egg is one of the key determinants of avian reproductive success, which may be compromised by microbial infections causing embryonic death. During incubation, outer eggshell bacterial communities pose a constant threat of pathogen translocation and embryo infection. One of the parental strategies to mitigate this threat is the incorporation of maternal immune factors into the egg albumen and yolk. It has been suggested that habitat changes like forest fragmentation can affect environmental factors and life-history traits that are linked to egg contamination. This study aims at investigating relationships between microbial pressure, immune investment and hatching success in two abundant forest bird species and analyzing to what extent these are driven by extrinsic (environmental) factors. We here compared (1) the bacterial load and composition on eggshells, (2) the level of immune defenses in eggs, and (3) the reproductive success between great (*Parus major*) and blue (*Cyanistes caeruleus*) tits in Belgium and examined if forest fragmentation affects these parameters. Analysis of 70 great tit and 34 blue tit eggshells revealed a similar microbiota composition (*Enterobacteriaceae*, *Lactobacillus* spp., *Firmicutes* and *Bacteroidetes*), but higher bacterial loads in great tits. Forest fragmentation was not identified as an important explanatory variable. Although a significant negative correlation between hatching success and bacterial load on the eggshells in great tits corroborates microbial pressure to be a driver of embryonic mortality, the overall hatching success was only marginally lower than in blue tits. This may be explained by the significantly higher levels of lysozyme and IgY in the eggs of great tits, protecting the embryo from increased infection pressure. Our results show that immune investment in eggs is suggested to be a species-specific adaptive trait that serves to protect hatchlings from pathogen pressure, which is not directly linked to habitat fragmentation.

## Introduction

Embryonic development of birds is a process that is threatened by microbial invasion [1]. Vertical transmission of pathogens during egg formation and horizontal transmission after oviposition may threaten the individual fitness and viability of the embryo and result in hatching failure [2]. Shortly after laying, the eggshell becomes susceptible to pathogen penetration [3]. As such, environmental factors such as nest materials, bacteria on the female’s skin, feathers and feces, nest visitors, and airborne bacteria are important risks of egg contamination [4].

To minimize embryonic contamination, the composition of the egg creates a natural physical barrier against bacterial penetration [5], and together with antimicrobial substances within the egg yolk and albumen [6], they constitute a first line of defense. In birds, females can influence the phenotype and fitness of their offspring by modifying the egg composition through the transfer of immunoglobulins (e.g. IgY) and antibacterial proteins to their eggs [7–8]. These maternal immune factors protect the embryo against bacteria which have succeeded in penetrating the eggshell, and the hatchling after the resorption of the remaining egg yolk and albumen. Amongst antimicrobial proteins in the egg albumen, lysozyme, ovotransferrin, and avidin are the three most abundant ones [9].

Several studies showed that the number of bacteria present on the eggshell is positively associated with the risk of trans-shell infection [1, 10]. Not only the bacterial load is a forerunner of hatching failure, but also the composition of the bacterial community and certainly the presence of pathogenic bacterial strains could play a role [11]. In the gastrointestinal tract of avian species, the phyla *Firmicutes* (including *Lactobacillus*) and *Bacteroidetes* and the family of *Enterobacteriaceae* are amongst the most abundant bacterial groups [12–13]. Most of the enteric bacteria have established a commensal status however, some members are also known as pathogens, especially *Enterobacteriaceae* such as *Escherichia coli*, *Salmonella* spp. and *Yersinia* spp., but also *Clostridium perfringens* belonging to the *Firmicutes* phylum [14].

Not only host and pathogen characteristics can influence embryonic development, but also the environment should be taken into account. Changes in host habitat, such as fragmentation of large, homogenous habitat blocks into small, isolated patches, can affect both extrinsic (environmental traits) and intrinsic (life-history traits) factors of the host which are linked to egg contamination and breeding performances. For instance, it has been described that brood parasitism is associated with increased bacterial contamination of host eggs [15–16] and that it occurs more in fragmented areas [17]. Habitat fragmentation also leads to changes in the social network and feeder visiting of birds [18], which is associated with pathogen acquisition and transmission [19]. Although some research has been performed on the effect of human encroachment on natural environments of wild bird populations and its effect on host-pathogen interactions, how extrinsic (environmental) drivers shape relationships between infection pressure, immune investment and breeding performance in forest birds, remains poorly known.

To fill this knowledge gap, this study aims at investigating these relationships in great and blue tits, two relatively closely-related forest species with strongly overlapping ecological niches that are widely distributed and abundant throughout Europe. We first examined bacterial infection pressure (load and community composition) on eggs of free-ranging blue and great tits in 19 mature deciduous forest fragments in East-Flanders (Belgium) and analyzed to what extent bacterial loads varied with fragment area. Next, we analyzed variation in maternal immune investment (IgY, avidin, lysozyme and ovotransferrin) into eggs and the extent to which this was correlated with hatching success.

## Materials and methods

### Study design and study site

**Fig 1:**
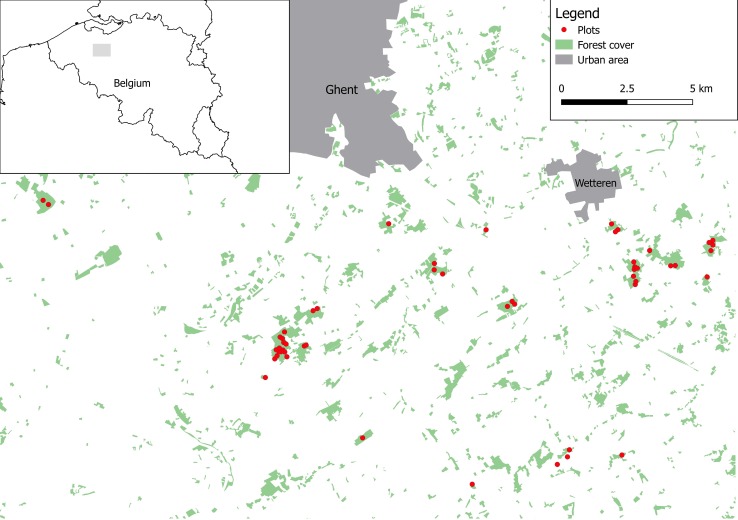
Map showing the location of all the study plots. The study of great and blue tit nests was performed in 53 study plots, established in 19 forest fragments.

We performed a study of blue and great tits in 53 study plots located in 19 mature (> 60 years) deciduous forest fragments in the south of Ghent (coordinates: 50°57'19"N, 3°43'31"E), northern Belgium (Fig 1; S1 Table). All study plots (30 x 30 m) were established in 2014 to study effects of tree species diversity and forest fragmentation on food web dynamics [20–21]. Forest fragments in which these plots were located, varied in size (range: 1.3 to 90.4 ha) and tree layer (3 focal species; Pedunculate oak (*Quercus robur*), Red oak (*Q*. *rubra*) and Beech (*Fagus sylvatica*) in monocultures, 2 species mixtures or 3 species mixtures). Surface area sizes for each forest fragment were calculated from detailed GIS layers.

During autumn 2014, 212 standard nest boxes for blue and great tits (dimensions 23 x 9 x 12 cm, entrance 32 mm) were installed at a height of 1.5 m, at each corner of a plot, of which 3 broke during the experiment [20]. During the breeding season of 2015, all nest boxes were checked at least twice a week to determine the total number of eggs produced (clutch size), possible brood parasitism (S1 Table) and the total number of hatchlings (S1 Fig).

Since fragmentation can lead to changes in feeder visiting, which is correlated with pathogen acquisition and transmission [18–19], it should be taken into account that the great and blue tits using these nest boxes had access and visited bird feeders in the gardens surrounding the study plots.

To avoid intraclutch variation, the fifth egg of each great and blue tit clutch was collected using sterile gloves, stored in a sterile bottle and transported to the laboratory where the eggs were cracked under a laminar flow cabinet. Egg yolk and egg white were collected and stored separately at -20°C. In order to avoid antimicrobial activity of the albumen, the inside of the eggshells was washed with sterile phosphate buffered saline (PBS) to remove the adhering egg albumen. The eggshell, including shell membranes, was transferred to an Eppendorf tube and stored at -20°C.

The percentage of hatching failure was calculated as ((1-(number of hatchlings/(clutch size– 1)))*100).

### Antimicrobial assays: Lysozyme, avidin and ovotransferrin

We assessed lysozyme concentrations following Ruuskanen et al. (2011) [22]. Briefly, albumen was diluted in phosphate buffer (67 mM, pH 6.2, dilution 1:500). A *Micrococcus lysodeikticus* (Sigma-Aldrich, Darmstadt, Germany) suspension was prepared in phosphate buffer (0.5 mg/ml). A hundred μl of the diluted albumen and 100 μl of the *Micrococcus* suspension were added to a 96 well plate (MaxiSorp Nunc-Immuno^TM^ plate, Thermo Fisher Scientific, Massachusetts, USA) and the absorbance was measured every 2 minutes, during 30 min at room temperature and at 450 nm using a Multiskan MS Reader (Labsystem Diagnostics Oy, Vantaa, Finland) with the Ascent Software, version 2.6. Each sample was analyzed in duplicate and before each measurement, the plate was mixed for 10 s. The results, given as Unit/mg protein, were calculated from the changes in absorbance per minute and compared to the standards (lysozyme from chicken egg white, Sigma-Aldrich).

To measure avidin, we used a modified version of the colorimetric method of Gan & Marquardt (1999) [23]. Therefore, each albumen sample was diluted 1:4 in carbonate–bicarbonate buffer (Sigma-Aldrich) and 100 μl of each 10-fold serial dilutions was added to a 96 well plate (MaxiSorp Nunc-Immuno plate), until a dilution factor of 11 was achieved. Serial dilutions of avidin (5–0.002 μg/ml, Sigma-Aldrich) were used as a standard. The plates were incubated at 4°C overnight and then rinsed 3 times with phosphate-buffered saline (PBS)/0.05% Tween-20 (Sigma-Aldrich). Superblock buffer (Pierce, Rockfords, USA) was added for 30 s at room temperature to prevent nonspecific binding. This was repeated twice. Subsequently, we added 100 μl of a 1:4000 dilution of biotin/horseradish peroxidase (Sigma-Aldrich) in Superblock/0.05% Tween-20 to each well. The plates were incubated for 25 min at room temperature, followed by a wash step with PBS/0.05% Tween-20. After washing the plate 5 times, 100 μl of blue peroxidase (POD) substrate (Roche, Reinach, Switzerland) were added to each well before incubating the plates at room temperature for 30 min. Finally, the absorbance was measured at 450 nm using a Multiskan MS Reader. The concentration of avidin (μg/ml) in each sample was calculated by comparison of absorbance values to those in the standard curve.

The concentration of ovotransferrin was determined using the total iron binding capacity assay of Yamanishi et al. (2002) [24]. Therefore, 125 μl of a 1:500 dilution of an iron-standard solution (1000 mg/ml; Sigma-Aldrich) in a buffer (pH 8.4) containing 300 mmol/l Tris (Thermo Fisher Scientific), 150 mmol/l sodium hydrogen carbonate (EMD Millipore, Darmstadt, Germany), and 4.2 g/l Triton X-100 (Sigma-Aldrich) was added to 24 μl of each albumen sample in wells of a 96-well plate (Nunc MaxiSorp). After 5 min of incubation at 37°C, a second reagent (pH 4.0) containing 10 mmol/l ferrozine (Baker, Maine, USA) and 32.6 mmol/l L-ascorbic acid (Thermo Fisher Scientific) in 50 mmol/l Tris buffer were added to each well and incubated at 37°C for 5 min. Subsequently 100 μl of a third reagent containing 600 mmol/l citric acid (Baker) and 25.6 mmol/l thiourea (Baker) was added. The absorbance was measured every 20 s at 570 and 660 nm for 6.2 min using Multiskan MS Reader. To calculate ovotransferrin concentration, we determined the difference in absorbance at 570/660 nm at the beginning and end of the 6.2-min period. The absolute ovotransferrin concentration (mg/ml) was calculated by comparing these values with those in a standard curve.

### Antibody titre analysis (IgY)

The antibody (IgY) level was determined using an indirect enzyme-linked immunosorbent assay (ELISA), modified from Morosinoto et al. (2013) [25]. Briefly, ELISA plates (MaxiSorp Nunc-Immuno plates) were coated overnight at 4°C with 50 μl anti-chicken IgG (produced in rabbit) diluted 1/2000 in carbonate coating buffer. Egg yolk was diluted 1/3 with distilled water and supernatant was collected after centrifugation at 13 000 rpm for 15 min (4°C). Subsequently, the supernatant diluted 1/2000 in 1% bovine serum albumin in phosphate buffered saline (BSA-PBS) was added to the wells (50 μl) and incubated for 3 hours at room temperature. An alkaline phosphatase conjugated rabbit anti-chicken IgY antibody (1/2000) (Sigma-Aldrich) was added overnight at 4°C as a secondary antibody. The plate was developed using p-nitrophenyl phosphate for 30 min. The optical density was measured at 405 nm using a Multiskan MS Reader.

### Bacteriological analysis: Enumeration of bacterial load by qPCR

DNA was extracted from the eggshell using a PowerLyzer® PowerSoil® DNA Isolation Kit (Qiagen, Venlo, The Netherlands) according to the manufacturer’s guidelines. The abundance of total bacteria, *Firmicutes* and *Bacteroidetes* phyla, *Enterobacteriaceae* family and *Lactobacillus* spp. were quantified using the primers and PCR protocols described in Table 1. Amplification and detection were performed using the CFX384 Bio-Rad Real-time PCR detection system (Bio-Rad, Nazareth, Belgium). Each reaction was done in duplicate in a 12-μl total reaction mixture using 2 x SensiMix SYBR No-ROX mix (Bioline, Luckenwalde, Germany) and 2 μl of DNA.

**Table 1:** Primers and qPCR protocols used to quantify the total bacteria, *Firmicutes*, *Enterobacteriaceae*, *Bacteroidetes* and *Lactobacillus* spp.

Bacterial groups	Reference	Primers (5' -3')	Primer concentration	PCR program
*Firmicutes*	[26]	F: GGAGYATGTGGTTTAATTCGAAGCA	0.5 μM	10' 95°C; (30'' 95°C, 30'' 60°C) x 40; 15'' 95°C
		R: AGCTGACGACAACCATGCAC	0.5 μM	
*Enterobacteriaceae*	[27]	F: CATTGACGTTACCCGCAGAAGAAGC	0.5 μM	10' 95°C; (30'' 95°C, 1' 63°C) x40; 15'' 95°C
		R: CTCTACGAGACTCAAGCTTGC	0.5 μM	
*Bacteroidetes*	[28]	F: CRAACAGGATTAGATACCCT	0.75 μM	10' 95°C; (15'' 95°C, 15'' 61.5°C, 20'' 72°C) x40; 15'' 95°C
		R: GGTAAGGTTCCTCGCGTAT	0.75 μM	
*Lactobacillus* spp.	[29]	F: GGAATCTTCCACAATGGACG	0.5 μM	20'' 95°C; (3''95°C, 30'' 57°C) x40; 15'' 95°C
		R: CGCTTTACGCCCAATAAATCCGG	0.5 μM	
Total bacteria	[30]	F: CGGYCCAGACTCCTACGGG	0.5 μM	10' 95°C; (1' 94°C, 1' 53°C, 2' 60°C) x 40;15'' 95°C
		R: TTACCGCGGCTGCTGGCAC	0.5 μM	

### Statistical analysis

First, in order to test whether total eggshell bacteria, *Firmicutes*, *Bacteroidetes*, *Enterobacteriaceae* and *Lactobacillus* numbers were influenced by fragment area, egg and nest characteristics (i.e. egg volume, clutch size and laying date) or species identity (i.e. great versus blue tit), linear mixed models (‘glmer’ function of R library ‘lme4’ [31] were run using bacteria counts as dependent variable. Forest fragment identity was included as a random effect to account for possible non-independence of nests within the same forest fragment, and models were run with a Poisson error distribution as bacterial loads were expressed as count data. We checked for overdispersion using the ‘c_hat’ function of R library ‘AICcmodavg’ [32], while significance of overdispersion estimates was assessed using the ‘overdisp_fun’ function available in 'PsychHelperFunctions’ library (https://rdrr.io/github/markushuff/PsychHelperFunctions). All c_hat variance inflation factors were larger than 1 (varying from 1.12 to 5.92) and were significant for all Poisson models considered (all P < 0.001). Therefore, an observation-level random effect was added in order to account for the overdispersion present in the data [33].

Second, in order to test whether bacterial eggshell communities significantly differed between great tits and blue tits, we applied an analysis of dissimilarity (ADONIS, as implemented in the R library ‘vegan’ [34]. Third, to compare concentrations of egg protein concentrations (i.e. concentrations of egg lysozyme, IgY, avidin and ovotransferrin) between both species, Gaussian linear mixed models were applied, using egg protein concentrations as dependent variable and species as explanatory variable while including forest fragment identity as a random effect. All model residuals were normally distributed (Shapiro-Wilk W > 0.90). Lastly, to explore the relationship between egg immune factors and reproductive success, hatching failure was modelled as a binominal process, comparing success against failure. All statistical tests were performed with R.

### Ethical considerations

This study was carried out in strict accordance with the recommendation in the European Convention for the Protection of Vertebrate Animals used for Experimental and other Scientific Purposes. All trapping and sampling protocols of blue and great tits were approved and permitted by the Ethical Committee VIB (the Flanders Institute for Biotechnology) Ghent site (EC2015-023).

## Results

### Bacterial abundance is higher on eggshells of great tits, but with a similar microbiota composition as in blue tits

**Fig 2:**
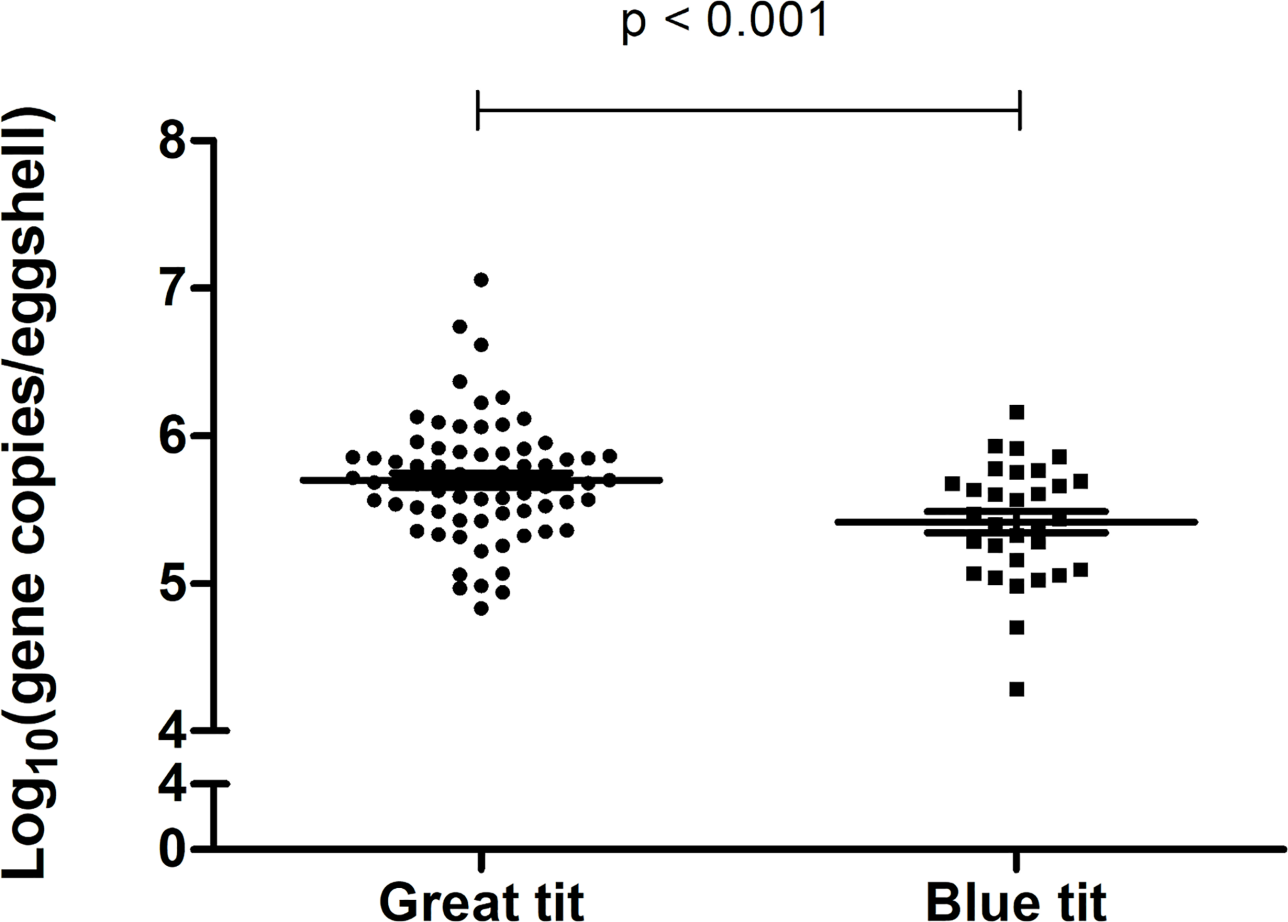
Number of total bacteria present on the eggshell of great and blue tits. The eggshells of great (n = 70; 42 different plots) and blue tits (n = 34; 25 plots) were analyzed using qPCR for bacterial presence. The results are expressed as the log_10_ of the copy number of the gene per eggshell. The whiskers represent the mean ± standard error of the mean. Statistical significance is shown by the p value.

**Fig 3:**
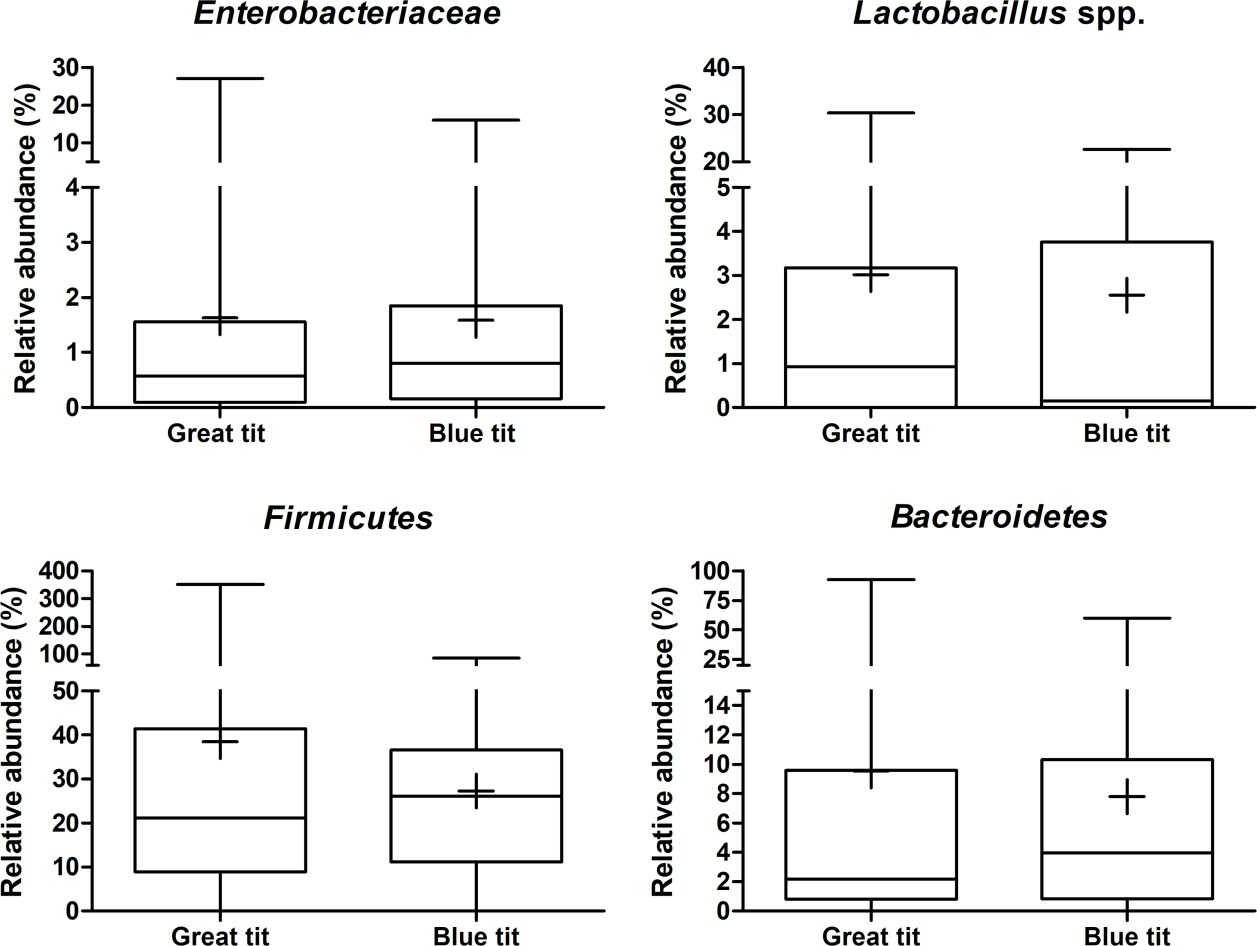
The relative abundance of bacterial groups present on the eggshell of great and blue tits. Box plots showing the relative abundance of *Enterobacteriaceae*, *Lactobacillus* spp., *Firmicutes* and *Bacteroidetes* compared to the total bacterial numbers present on the eggshell of great and blue tits, as assessed with qPCR. The whiskers represent the median, the minimum and maximum values, and the first and third quartiles. The plus indicates the mean value.

The bacterial loads of 70 eggs of great tits collected from nest boxes in 42 plots, and of 34 eggs of blue tits collected in nests from 25 plots (summarized in S1 Table) were determined by qPCR. Bacterial loads were higher (p < 0.001) on the eggshells of great tits compared to those on the shells of blue tits (Fig 2 and S2 Table). The mean (± SE) eggshell total bacterial count (gene copies / eggshell) of great and blue tit eggs was 8.57 x 10^5^ ± 1.83 x 10^5^ and 3.67 x 10^5^ ± 5.49 x 10^4^, respectively. This corresponds to a Log_10_ value of 5.70 ± 0.05 for great tits and 5.42 ± 0.07 for blue tits (Table 2). Fragment area, egg volume, laying date and clutch size could not explain the pattern of bacterial load on eggs of great and blue tits (all p > 0.05; S3 Table). Although more bacteria were present on the eggshells of great tits, no significant differences were observed in the relative abundance of the composition of eggshell microbiota (Fig 3). Similar proportions (%) of *Enterobacteriaceae*, *Lactobacillus* spp., *Firmicutes*, and *Bacteroidetes* were observed relative to the total amount of bacteria present on the eggshell of both bird species (Table 2 and S2 Table).

**Table 2:** Difference in bacterial load, microbiota composition, IgY and presence of antibacterial proteins between blue and great tits. Shown is the mean ± SE of the total eggshell bacterial load, the proportion (in %) of *Enterobacteriaceae*, *Lactobacillus* spp., *Firmicutes*, and *Bacteroidetes* and egg immune factors of blue and great tit eggs.

Measurement	Blue tit	Great tit
Eggshell total bacterial count (gene copies/eggshell)	3.67 x 10^5^ ± 5.49 x 10^4^	8.57 x 10^5^ ± 1.83 x 10^5^
Log10 eggshell total bacterial count (gene copies/eggshell)	5.42 ± 0.07	5.70 ± 0.05
Proportion of *Enterobacteriaceae* (%)	1.6 ± 0.49	1.6 ± 0.43
Proportion of *Lactobacillus* spp. (%)	2.5 ± 0.79	3.0 ± 0.68
Proportion of *Firmicutes* (%)	27.3 ± 3.90	38.5 ± 7.38
Proportion of *Bacteroidetes* (%)	7.8 ± 1.99	9.5 ± 2.17
IgY (OD)	0.48 ± 0.037	0.74 ± 0.036
Lysozyme (unit/mg)	68675.56 ± 5878.35	107952.81 ± 3991.08
Avidin (μg/ml)	0.23 ± 0.035	0.32 ± 0.034
Ovotransferrin (mg/ml)	3.63 ± 0.46	4.34 ± 0.35

### Higher immune factor concentrations in eggs of great tits

**Fig 4:**
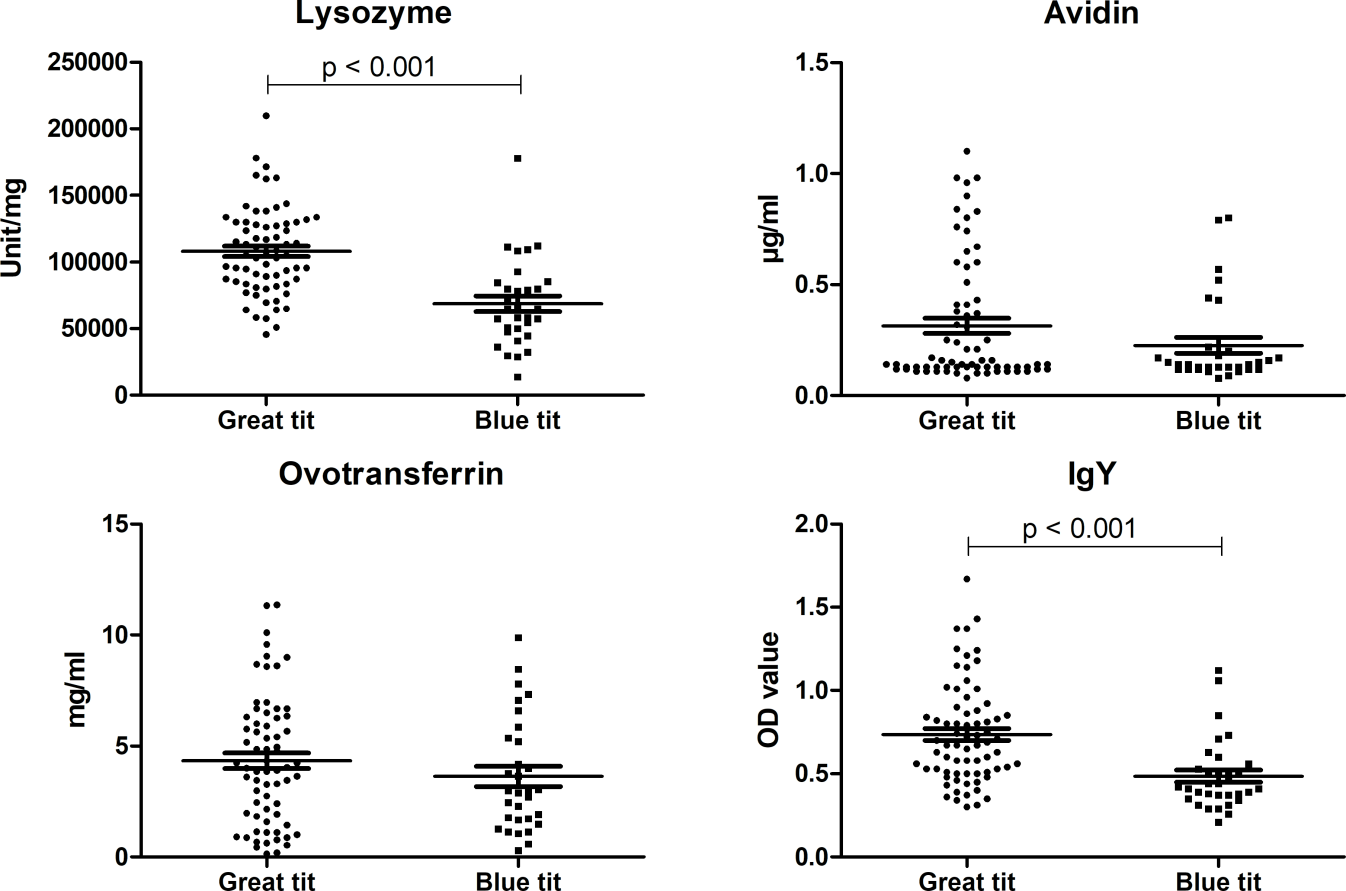
Concentration of antimicrobial proteins and IgY antibodies. Lysozyme, avidin, ovotransferrin and IgY were determined in the eggs of great and blue tits. The results are expressed as unit/mg lysozyme, μg/ml avidin, mg/ml ovotransferrin or as OD value for IgY. The whiskers represent the mean ± standard error of the mean. Statistical significance is shown by the p value.

Egg lysozyme and IgY levels were significantly (p < 0.001) higher in egg albumen and egg yolk of great tits compared to blue tits (Fig 4, Table 2 and S2 Table). Mean (± SE) concentrations of lysozyme and IgY were 68675.56 ± 5878.35 and 0.48 ± 0.037 in blue tits and 107952.81 ± 3991.08 and 0.74 ± 0.036 in great tits. These differences are species specific as “species” was shown to be a driver for IgY and lysozyme concentrations (p < 0.001; S4 and S5 Tables). Great tits eggs also tended to show higher concentrations of avidin and ovotransferrin, but without reaching statistical significance (p > 0.05; Table 2 and S2 Table).

### Hatching success in great tits is only slightly impacted by the increased microbial pressure

**Fig 5:**
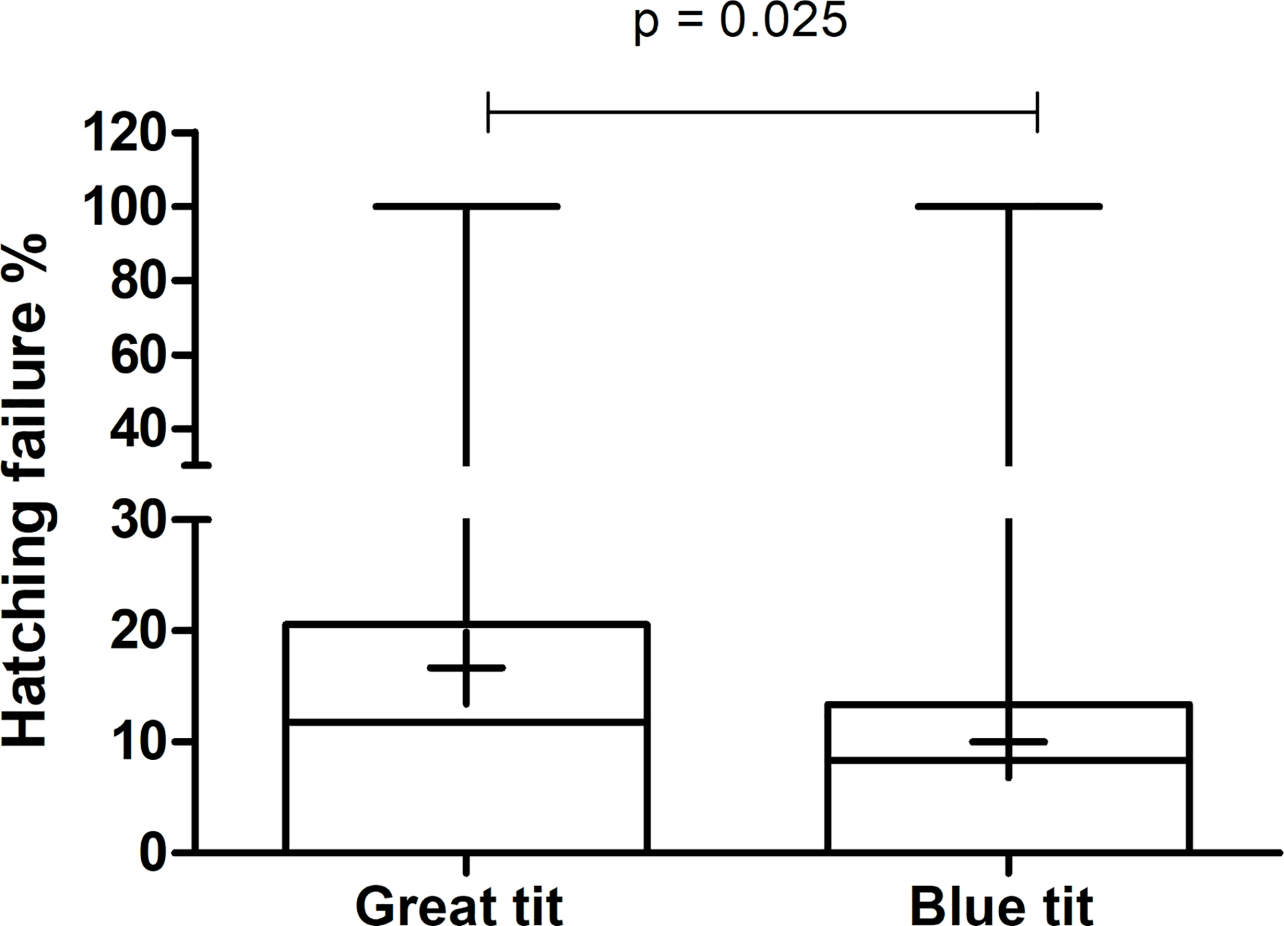
Hatching failure in nests of great and blue tits. Shown is the percentage of hatching failure. The whiskers represent the median, the minimum and maximum values, and the first and third quartiles. The plus indicates the mean value. Statistical significance is shown by the p value.

Hatching success in clutches of great tits declined with increasing bacterial load of the fifth egg (p = 0.024; S6 Table). In blue tits, none of the studied variables were found to correlate with hatching success. At species level, hatching failure was significantly higher in great tits (16.66 ± 2.79) than in blue tits (10.02 ± 2.92%) (p = 0.025; Fig 5 and S2 Table).

## Discussion

We provide evidence for a higher infection pressure in great tit eggs than in those of the sympatric, ecologically similar blue tit, while the bacterial load of neither species was associated with variation in fragment area, egg volume, laying date or clutch size.

Avian species are known to apply different behavioral, chemical and physical strategies to control embryo infections, such as the use of intrinsic properties of plants to protect their nestlings against contamination with parasites and pathogens. Among passerines, blue tits have been reported to use aromatic plants as nest materials, possibly exploiting the antimicrobial properties of essential oils [35]. In our study, we detected leaves of the aromatic plant *Stachys sylvatica*, and pine needles in a number of blue tit nests, and essential oils of both plant species are believed to have antimicrobial activities [36–37]. As no such leaves or needles were detected in great tit nests, differential use of nest material may partly explain the observed differences in microbial pressure between both species. Alternatively, or in addition, differential bacterial accumulation may result from differences in nest sanitization, and results of our study would point towards a higher nest hygiene in blue tits. However, this hypothesis contradicts the results of Goodenough & Stallwood (2010) showing higher bacterial loads in blue tit nests than in those of great tits, hence more empirical studies are needed to test this hypothesis [38].

In contrast to bacterial load, relative egg microbiota composition (*Enterobacteriaceae*, *Lactobacillus* spp., *Firmicutes* and *Bacteroidetes*) did not differ between great and blue tits, with the *Firmicutes* phylum being the most abundant in both species. Such pattern is in line with gastrointestinal microbiota sampled in adults from various bird species [13], supporting the hypothesis that bacteria are transmitted from the female cloaca to the eggs [11]. Although most members of these bacterial groups are commensals, several bacterial species are also known as primary or opportunistic pathogens. Especially bacteria of the *Enterobacteriaceae* family such as *E*. *coli*, *Salmonella*, *Yersinia*, *Klebsiella*, *Citrobacter* and *Enterobacter* have been reported to cause disease and mortality in nestling passerines [39]. Additionally, *Streptococcaceae* of the phylum *Firmicutes* has been reported to cause embryonic death and infections in nestlings [2].

Transmission of antimicrobials and IgY antibodies to the egg constitutes an important chemical defense mechanism in birds. Lysozyme catalyzes the lysis of cell walls of gram-positive bacteria and plays an important role pre-hatching, whereas IgY antibodies particularly protect nestling post-hatching. While still fairly speculative, some authors suggested that mothers may distribute antimicrobial proteins differentially within and among clutches [6], based on food availability [40] and depending on male attractiveness [9]. These studies also provide evidence that birds may have evolved to differentially transmitting antimicrobials to increase the probability of offspring survival. In our study, great tits incorporated more lysozyme and IgY into the eggs than blue tits, suggesting that great tit females may manipulate their antimicrobial allocation to compensate for the higher pathogen load. Or, another speculative possibility is that great tits are generally more efficient in fighting microbes, through which they can select for behaviour and/or nest environments that are associated with higher infection probabilities. The increased lysozyme and IgY allocation may however explain why the reproductive success of great tits was only moderately lower than that of blue tits, despite their larger infection pressure and the observed correlation between infection pressure and hatching failure.

Summarized, the results obtained by this study show that although great and blue tits are relatively closely related and ecologically similar, eggs of great tits are exposed to higher microbial pressures. Great tit eggs contain more lysozyme and IgY, which could limit the negative effect of pathogen pressure on reproductive parameters.

## Supporting information

S1 Fig Overview of the clutch size and the number of hatchlings in the nests of great and blue tits. (TIF)

S1 Table Summary of sampled great (PM) and blue (PC) tit eggs in the different study plots. (PDF)

S2 Table Summary of statistical analyses examining the difference between blue and great tits. (PDF)

S3 Table Summary of statistical analyses examining the driving factors for eggshell bacterial loads. (PDF)

S4 Table Summary of statistical analyses examining the driving factors for lysozyme allocation. (PDF)

S5 Table Summary of statistical analyses examining the driving factors for IgY allocation. (PDF)

S6 Table Summary of statistical analyses examining the driving factors for hatching failure. (PDF)

## References

[1] Cook MI, Beissinger SR, Toranzos GA, Rodriguez RA, Arendt WJ. Trans–shell infection by pathogenic micro–organisms reduces the shelf life of non–incubated bird's eggs: a constraint on the onset of incubation? P Roy Soc Lond B Bio. 2003;270: 2233–2240. 10.1098/rspb.2003.2508 14613609 PMC1691504

[2] Pinowski J, Barkowska M, Kruszewicz A, Kruszewicz A. The causes of the mortality of eggs and nestlings of Passer sp. J Biosciences. 1994;19: 441–451. 10.1007/BF02703180

[3] Gantois I, Ducatelle R, Pasmans F, Haesebrouck F, Gast R, Humphrey TJ, et al Mechanisms of egg contamination by *Salmonella* Enteritidis. FEMS Microbiol Rev. 2009;33: 718–738. 10.1111/j.1574-6976.2008.00161.x 19207743

[4] Baggott G, Graeme-Cook K. Microbiology of natural incubation In: Deeming DC, editor. Oxford Ornithology Series: 2002 pp. 179–188.

[5] Sparks N. Shell accessory materials: structure and function In: Board RG, Fuller R, editors. Microbiology of the Avian Egg: 1994 pp. 25–42.

[6] Shawkey MD, Kosciuch KL, Liu M, Rohwer FC, Loos ER, Wang JM, et al Do birds differentially distribute antimicrobial proteins within clutches of eggs? Behav Ecol. 2008;19: 920–927. 10.1093/beheco/arn019

[7] Board R, Tranter H. The microbiology of eggs In: Stadelman WJ, Cotterill OJ, editors. Egg science and technology: 1995 pp. 81–104.

[8] Mousseau TA, Fox CW. The adaptive significance of maternal effects. Trends Ecol Evol. 1998;13: 403–407. 10.1016/S0169-5347(98)01472-4 21238360

[9] D’Alba L, Shawkey MD, Korsten P, Vedder O, Kingma SA, Komdeur J, et al Differential deposition of antimicrobial proteins in blue tit (*Cyanistes caeruleus*) clutches by laying order and male attractiveness. Behav Ecol Sociobiol. 2010;64: 1037–1045. 10.1007/s00265-010-0919-y 20414331 PMC2854352

[10] Shawkey MD, Firestone MK, Brodie EL, Beissinger SR. Avian incubation inhibits growth and diversification of bacterial assemblages on eggs. PLoS One. 2009;4: e4522 10.1371/journal.pone.0004522 19225566 PMC2639702

[11] Ruiz‐de‐Castañeda R, Vela AI, Lobato E, Briones V, Moreno J. Prevalence of potentially pathogenic culturable bacteria on eggshells and in cloacae of female Pied Flycatchers in a temperate habitat in central Spain. J Field Ornithol. 2011;82: 215–224. 10.1111/j.1557-9263.2011.00324.x

[12] De Maesschalck C, Eeckhaut V, Maertens L, De Lange L, Marchal L, Nezer C, et al Effects of xylo-oligosaccharides on broiler chicken performance and microbiota. Appl Environ Microb. 2015;81: 5880–5888. 10.1128/AEM.01616-15 26092452 PMC4551243

[13] Garcia-Mazcorro JF, Castillo-Carranza SA, Guard B, Gomez-Vazquez JP, Dowd SE, Brigthsmith DJ. Comprehensive molecular characterization of bacterial communities in feces of pet birds using 16S marker sequencing. Microb Ecol. 2017;73: 224–235. 10.1007/s00248-016-0840-7 27568186

[14] Rogers KH. Prevalence of pathogenic enteric bacteria in wild birds associated with agriculture in Humboldt County, California. M. Sc. Thesis, Humboldt State University. 2006. Avialable from: http://humboldt-dspace.calstate.edu/handle/2148/100

[15] Soler J, Peralta-Sánchez J, Martínez‐Bueno M, Martín-Vivaldi M, Martín‐Gálvez D, Vela AI, et al Brood parasitism is associated with increased bacterial contamination of host eggs: bacterial loads of host and parasitic eggs. Biol J Linn Soc. 2011;103: 836–848. 10.1007/s00114-011-0830-z

[16] Barrientos R, Bueno-Enciso J, Serrano-Davies E, Sanz JJ. Facultative interspecific brood parasitism in tits: a last resort to coping with nest-hole shortage. Behav Ecol Sociobiol. 2015;69: 1603–1615. 10.1007/s00265-015-1972-3

[17] Borges FJA, Marini MÂ. Birds nesting survival in disturbed and protected Neotropical savannas. Biodivers Conserv. 2010;19: 223–236. 10.1007/s10531-009-9718-z

[18] Latimer CE, Cooper SJ, Karasov WH, Zuckerberg B. Does habitat fragmentation promote climate‐resilient phenotypes? Oikos. 2018 10.1111/oik.05111

[19] Adelman JS, Moyers SC, Farine DR, Hawley DM. Feeder use predicts both acquisition and transmission of a contagious pathogen in a North American songbird. P Roy Soc Lond B Bio. 2015;282:1429 10.1098/rspb.2015.1429 26378215 PMC4614752

[20] De Groote SR, van Schrojenstein L, Irene M, Sercu BK, Dekeukeleire D, Boonyarittichaikij R, et al Tree species identity outweighs the effects of tree species diversity and forest fragmentation on understorey diversity and composition. Plant Ecol Evol. 2017;150: 229–239. 10.5091/plecevo.2017.1331

[21] Boonyarittichaikij R, Verbrugghe E, Dekeukeleire D, De Beelde R, Rouffaer L, Haesendonck R, et al *Salmonella* Typhimurium DT193 and DT99 are present in great and blue tits in Flanders, Belgium. PloS One. 2017;12: e0187640 10.1371/journal.pone.0187640 29112955 PMC5675436

[22] Ruuskanen S, Siitari H, Eeva T, Belskii E, Järvinen A, Kerimov, et al (2011). Geographical variation in egg mass and egg content in a passerine bird. PloS One. 2011;6: e25360 10.1371/journal.pone.0025360 22110579 PMC3215694

[23] Gan Z, Marquardt RR. Colorimetric competitive inhibition method for the quantitation of avidin, streptavidin and biotin. J Biochem Bioph Meth. 1999;39: 1–6. 10.1016/S0165-022X(98)00051-7 10344497

[24] Yamanishi H, Iyama S, Yamaguchi Y, Kanakura Y, Iwatani Y. Modification of fully automated total iron-binding capacity (TIBC) assay in serum and comparison with dimension TIBC method. Clin Chem. 2002;48: 1565–1570. 12194935

[25] Morosinotto C, Ruuskanen S, Thomson RL, Siitari H, Korpimäki E, Laaksonen T. Predation risk affects the levels of maternal immune factors in avian eggs. J Avian Biol. 2013;44: 427–436. 10.1111/j.1600-048X.2013.00084.x

[26] Guo X, Xia X, Tang R, Zhou J, Zhao H, Wang K. Development of a real‐time PCR method for *Firmicutes* and Bacteroidetes in faeces and its application to quantify intestinal population of obese and lean pigs. Lett Appl Microbiol. 2008;47: 367–373. 10.1111/j.1472-765X.2008.02408.x 19146523

[27] Bartosch S, Fite A, Macfarlane GT, McMurdo ME. Characterization of bacterial communities in feces from healthy elderly volunteers and hospitalized elderly patients by using real-time PCR and effects of antibiotic treatment on the fecal microbiota. Appl Environ Microb. 2004;70: 3575–3581. 10.1128/AEM.70.6.3575–3581.2004 PMC427772 15184159

[28] De Gregoris TB, Aldred N, Clare AS, Burgess JG. Improvement of phylum-and class-specific primers for real-time PCR quantification of bacterial taxa. J Microbiol Meth. 2011;86: 351–356. 10.1016/j.mimet.2011.06.010 21704084

[29] Abdulamir AS, Yoke TS, Nordin N, Bakar FA. Detection and quantification of probiotic bacteria using optimized DNA extraction, traditional and real-time PCR methods in complex microbial communities. Afr J Biotechnol. 2010;9: 1481–1492.

[30] Lee DH, Zo YG, Kim SJ. Nonradioactive method to study genetic profiles of natural bacterial communities by PCR-single-strand-conformation polymorphism. Appl Environ Microbiol. 1996;62: 3112–3120. 8795197 10.1128/aem.62.9.3112-3120.1996 PMC168103

[31] Bates D, Maechler M, Bolker B, Walker S. Fitting Linear Mixed-Effects Models Using lme4. J Stat Softw. 2015;67: 1–48. doi: 10.18637/jss.v067.i01

[32] Mazerolle MJ. AICcmodavg: Model selection and multimodel inference based on (Q)AIC(c). 2017. R package version 2.1–1. Available from: https://cran.r-project.org/package=AICcmodavg

[33] Harrison XA. Using observation-level random effects to model overdispersion in count data in ecology and evolution. PeerJ. 2014;2: e616 10.7717/peerj.616 25320683 PMC4194460

[34] Oksanen J, Guillaume Blanchet F, Friendly M, Kindt R, Legendre P, McGlinn D, et al. vegan: Community Ecology Package. R package version 2.4–3. 2017. Available from: https://CRAN.R-project.org/package=vegan

[35] Mennerat A, Mirleau P, Blondel J, Perret P, Lambrechts MM, Heeb P. Aromatic plants in nests of the blue tit Cyanistes caeruleus protect chicks from bacteria. Oecologia. 2009;161, 849–855. 10.1007/s00442-009-1418-6 19633988

[36] Skaltsa HD, Demetzos C, Lazari D, Sokovic M. Essential oil analysis and antimicrobial activity of eight Stachys species from Greece. Phytochemistry. 2003;64: 743–752. 10.1016/S0031-9422(03)00386-8 13679097

[37] Zeng WC, Zhang Z, Gao H, Jia LR, He Q. Chemical composition, antioxidant, and antimicrobial activities of essential oil from pine needle (Cedrus deodara). J Food Sci. 2012;77: C824–9. 10.1111/j.1750-3841.2012.02767.x 22757704

[38] Goodenough AE, Stallwood B. Intraspecific variation and interspecific differences in the bacterial and fungal assemblages of blue tit (*Cyanistes caeruleus*) and great tit (*Parus major*) nests. Microb Ecol. 2010;59: 221–232. 10.1007/s00248-009-9591-z 19830477

[39] Dorrestein GM. Bacterial and parasitic diseases of passerines. Vet Clin North Am Exot Anim Pract. 2009;12: 433–451. 10.1016/j.cvex.2009.07.005 19732703

[40] Pihlaja M, Siitari H, Alatalo RV. Maternal antibodies in a wild altricial bird: effects on offspring immunity, growth and survival. J Anim Ecol. 2006;75: 1154–1164. 10.1111/j.1365-2656.2006.01136.x 16922851

