# Supplementary figures and images for: Mitigating the impact of microbial pressure on great (Parus major) and blue (Cyanistes caeruleus) tit hatching success through maternal immune investment
Source: PLoS One. 2018 Oct 4;13(10):e0204022. doi: 10.1371/journal.pone.0204022 (PMC6171831; doi:10.1371/journal.pone.0204022)

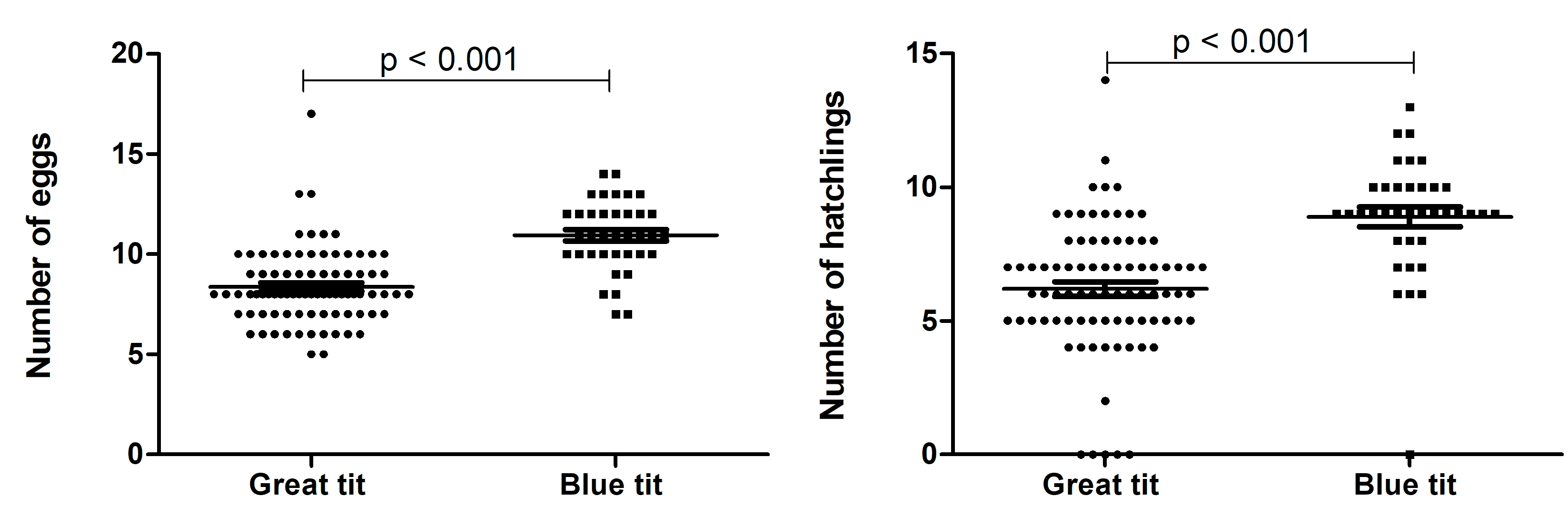

Supplement: S1 Fig — (TIF) [file pone.0204022.s001.tif]
