# Supplementary material for: Mitigating the impact of microbial pressure on great (Parus major) and blue (Cyanistes caeruleus) tit hatching success through maternal immune investment
Source: PLoS One. 2018 Oct 4;13(10):e0204022. doi: 10.1371/journal.pone.0204022 (PMC6171831; doi:10.1371/journal.pone.0204022)
Supplement: S1 Table — (PDF) [file pone.0204022.s002.pdf]

Forest	Plot	Latitude	Longitude	Surface area (ha)	tree diversity (spp.)	Nestbox	spp.
Lemberge	1	50.990342	3.7732	16.68	2	1.2	PM
						1.3	PC
						1.4	PM
Nerenbos (Merelbeke)	2	50.961391	3.73474	41.74	2	2.1	PC
						2.2	PC
						2.3	PM
						2.4	PM
	3	50.960503	3.73237	41.74	2	3.2	PM
						3.4	PC
Heilig Geestgoed (Merelbeke)	4	50.948551	3.728218	27.49	2	4.1	PM
						4.3	PM
	5	50.948747	3.728979	27.49	2	5.1	PM
						5.2	PM
Makegembos (Merelbeke)	7	50.951324	3.715364	83.77	2	7.1	PM
						7.2	PC
						7.3	PM
	8	50.950905	3.716254	83.77	1	8.2	PC
	9	50.949559	3.716739	83.77	1	9.1	PC
						9.3	PM
						9.4	PM
	10	50.94883	3.718443	83.77	1	10.3	PM
Harentbeekbos (Merelbeke)	12	50.9464	3.717465	83.77	1	12.1	PC
						12.3	PM
						12.4	PM
	13	50.946545	3.715697	83.77	1	13.2	PC
						13.3	PC*
	16	50.947438	3.714835	83.77	1	16.1	PM
						16.2	PC
						16.4	PM
	17	50.944474	3.718904	83.77	2	17.1	PC
						17.2	PM
						17.3	PC
	18	50.943761	3.712352	83.77	1	18.1	PM
						18.2	PM
						18.3	PM
						18.4	PM
	19	50.9449	3.713682	83.77	1	19.3	PM
						19.4	PC
Wannegatstraat (Gavere)	20	50.937592	3.707042	3.03	1	20.1	PM
						20.2	PC

						20.3	PC
Bueren (Melle)	21	50.9886	3.82614	6.19	2	21.1	PC
						21.2	PM
Aalmoezenijbos (Oosterzele)	22	50.976081	3.798739	23.57	2	22.1	PM
						22.2	PM
						22.4	PC
	23	50.974748	3.797965	23.57	2	23.1	PM
						23.3	PM
	24	50.973663	3.802786	23.57	1	24.1	PM
						24.2	PM
						24.4	PM
Spiegeldries bos (Oosterzele)	25	50.916874	3.760309	11.37	2	25.1	PM
						25.2	PM
						25.3	PM
Zottegem	26	50.901508	3.819877	3.53	3	26.2	PM
						26.3	PM
						26.4	PM
St-Lievens-Houtem	27	50.908521	3.865333	1.31	2	27.4	PM
	28	50.911148	3.871161	1.59	2	28.2	PC
						28.3	PC
						28.4	PM
	29	50.913516	3.872813	5.63	1	29.2	PC
						29.4	PM
	30	50.91155	3.901021	12.04	1	30.1	PC
						30.3	PM
						30.4	PC
	31	50.973112	3.946005	9.21	1	31.3	PM
Nonnenbos (Serskamp)	32	50.985475	3.949129	32.69	2	32.2	PM
Serskamp	36	50.976824	3.926348	58.9	2	36.2	PC
						36.3	PM
						36.4	PC
	37	50.976977	3.9288	58.9	2	37.3	PM
						37.4	PM
Oud smetlede	38	50.978195	3.906263	47.77	1	38.1	PM
						38.3	PM
						38.4	PM
	39	50.976306	3.907334	47.77	1	39.4	PM
	40	50.975601	3.906863	47.77	2	40.1	PM
	41	50.976082	3.908319	47.77	3	41.3	PM
	43	50.970567	3.907196	47.77	1	43.1	PC
						43.2	PM

						43.3	PM
	44	50.971339	3.907868	47.77	1	44.1	PM
						44.3	PM
	45	50.982	3.914797	58.9	2	45.2	PM*
Hospicebossen (Nazareth)	46	50.99087	3.894436	18.73	1	46.3	PC
	47	50.98917	3.897568	18.73	2	47.1	PM
						47.2	PM
	48	50.988468	3.89644	18.73	1	48.1	PC
						48.2	PM
						48.4	PM
Oosterzele	49	50.962551	3.838403	30.65	1	49.1	PM
						49.3	PM
						49.4	PC
	50	50.96349	3.842156	30.65	3	50.1	PC
						50.2	PM
						50.3	PC
						50.4	PM
	51	50.964019	3.840559	30.65	1	51.1	PM
						51.2	PC
						51.4	PC
Ooidonk (Deinze)	52	50.996011	3.588524	46.16	1	52.2	PM
	53	50.997431	3.585583	46.16	3	53.1	PC
						53.4	PM

An asterisk (*) indicates a nest where facultative brood parasitism between the two tit species was observed.
