# Supplementary material for: Mitigating the impact of microbial pressure on great (Parus major) and blue (Cyanistes caeruleus) tit hatching success through maternal immune investment
Source: PLoS One. 2018 Oct 4;13(10):e0204022. doi: 10.1371/journal.pone.0204022 (PMC6171831; doi:10.1371/journal.pone.0204022)
Supplement: S2 Table — (PDF) [file pone.0204022.s003.pdf]

Factors	t/z/f-value	p-value
Log_{10} eggshell bacterial load	3.685	0.000373
Enterobacteriaceae	1.515	0.130
Lactobacillus spp.	1.456	0.145
Firmicutes	0.966	0.334
Bacteroidetes	1.356	0.175
Lysozyme	5.768	8.2×10^{-9}
Avidin	0.842	0.402
Ovotransferrin	1.131	0.261
IgY	4.598	1.26×10^{-5}
Hatching failure	-2.241	0.025
