# Supplementary material for: Mitigating the impact of microbial pressure on great (Parus major) and blue (Cyanistes caeruleus) tit hatching success through maternal immune investment
Source: PLoS One. 2018 Oct 4;13(10):e0204022. doi: 10.1371/journal.pone.0204022 (PMC6171831; doi:10.1371/journal.pone.0204022)
Supplement: S3 Table — (PDF) [file pone.0204022.s004.pdf]

Factors	Great tit		Blue tit	
	z-value	p-value	z-value	p-value
Forest fragment surface area	-0.10	0.917	1.31	0.191
Egg volume	-0.04	0.970	-0.27	0.785
Clutch size	-0.07	0.940	-1.94	0.065
Laying date	0.50	0.614	1.31	0.191
