# Supplementary material for: Mitigating the impact of microbial pressure on great (Parus major) and blue (Cyanistes caeruleus) tit hatching success through maternal immune investment
Source: PLoS One. 2018 Oct 4;13(10):e0204022. doi: 10.1371/journal.pone.0204022 (PMC6171831; doi:10.1371/journal.pone.0204022)
Supplement: S5 Table — (PDF) [file pone.0204022.s006.pdf]

Factors	t-value	p-value
Eggshell bacterial load	-0.601	0.549
Species	4.058	0.000103
