# Supplementary material for: Mitigating the impact of microbial pressure on great (Parus major) and blue (Cyanistes caeruleus) tit hatching success through maternal immune investment
Source: PLoS One. 2018 Oct 4;13(10):e0204022. doi: 10.1371/journal.pone.0204022 (PMC6171831; doi:10.1371/journal.pone.0204022)
Supplement: S6 Table — (PDF) [file pone.0204022.s007.pdf]

Factors	Great tit		Blue tit	
	z-value	p-value	z-value	p-value
Forest fragment surface area	0.715	0.474	0.961	0.337
Eggshell bacterial load	-2.262	0.024	1.562	0.118
Enterobacteriaceae	-0.874	0.382	1.045	0.296
Lactobacillus spp.	0.686	0.493	-0.082	0.935
Firmicutes	1.39	0.165	1.729	0.839
Bacteroidetes	1.23	0.219	-0.846	0.398
Lysozyme	-1.47	0.143	-1.492	0.136
Avidin	0.037	0.970	0.170	0.865
Ovotransferrin	0.438	0.662	0.877	0.380
IgY	1.167	0.243	0.017	0.987
Clutch size	1.149	0.250	-0.232	0.817
Laying date	-0.299	0.767	0.350	0.727
